# Effects of Antimicrobial Flavonoids Against Representative Bacteria and Fungi: A Review of the Literature

**DOI:** 10.7759/cureus.62765

**Published:** 2024-06-20

**Authors:** Mohamed E Hamid, Fares Alamri, Ihab M Abdelrahim, Martin Joseph, Maria M Elamin, Alhafez M Alraih

**Affiliations:** 1 Department of Clinical Microbiology and Parasitology, College of Medicine, King Khalid University, Abha, SAU; 2 Mawhiba Academic Enrichment Program, King Khalid University, Abha, SAU; 3 Department of Clinical Microbiology and Parasitology,, Faculty of Medicine, King Khalid University, KSA, abha, SAU; 4 Faculty of Pharmacy, National University, Khartoum, Sudan, SDN; 5 Department of Chemistry, College of Science and Arts, King Khalid University, Mohail Aseer, SAU

**Keywords:** food elements, yeast, bacteria, antimicrobial, flavonoids

## Abstract

Introduction: Effective medications are becoming more necessary to combat the global rise in antimicrobial resistance. The findings that some flavonoids have antibacterial properties have urged interest in flavonoid research. The aim of this work was to investigate the inhibitory properties of fisetin, fisetinidin, 7,3`,4`-trihydroxyflavone (THF), and 7,3`,4`-trihydroxyflavonol (THF-) against selected pathogenic bacteria and fungi and to review the literature on relevant compounds.

Methods: An in vitro experiment was performed on 19 organisms (gram-positive, gram-negative, and yeast fungi) using varying concentrations (100-1000 ug/mL) of fisetin, fisetinidin, THF, and THF-. Using the agar well diffusion method. The in vitro activity of flavonoid compounds against gram-positive, gram-negative, and yeasts was assessed using a serial dilution of the four compounds against organism suspensions (50 µL of 0.5 McFarland). Inoculated agar plates were incubated aerobically at 37^o^C. The results of inhibition were recorded after 24, 48, and 72 hours.

Results: Various classes of flavonoids from different sources have been reviewed for their antimicrobial effects. They showed various inhibitory reactions against a plethora of gram-positive, gram-negative, and yeast organisms. In the present study, the selected four compounds have shown varying antibacterial effects, as have the reviewed flavonoids from the literature. With minimum inhibitory concentrations (MICs) ranging from 100 ug/mL to 1000 ug/mL, the substances fisetin, fisetinidin, THF, and THF- demonstrated inhibitory action against the examined species. The main activity was against *Staphylococcus*, *Bacillus*, *Acinetobacter*, *Proteus*, and *Pseudomonas *species. Fisetin and fisetinidin did not inhibit *Escherichia coli*, whereas THF and THF- exhibited inhibitory action.

Conclusions: Flavonoids, a readily accessible dietary ingredient, remain a viable treatment option for infectious diseases. This study suggests that THF, THF-, fisetin, and fisetinidin may be helpful in stopping the growth of some pathogens, especially staphylococci. Improvements to flavonoids' pharmacokinetics and diffusion may encourage their use in therapy as an adjuvant to conventional medications.

## Introduction

Flavonoids are an important class of secondary metabolites that are widely distributed throughout the tissues and organs of a wide variety of plant species. They make up a significant portion of the human diet. Among them are grains, fruits, vegetables, herbs, flowers, nuts, stems, and seeds. To date, almost 10,000 distinct flavonoid compounds have been identified [1, 2].

Numerous studies have shown that including flavonoids in a healthy diet may have benefits as antimicrobial agents [2, 3]. Polyphenols have an effect on some bacterial strains from different species, such as *Actinomyces viscosus, Escherichia coli, Staphylococcus aureus, Streptococcus mutans*, and *Streptococcus sanguinis *[4]. A review of the literature indicates that flavonoids inhibit the synthesis of cell envelopes, virulence factors, efflux pumps, membrane rupture, biofilms, nucleic acid synthesis, and bacterial motility [5]. Effective medications are becoming more and more necessary to treat microbial illnesses and combat antibiotic resistance. Given that most synthetic antimicrobials have developed resistance in recent years, natural remedies might provide more potent substitutes to address this need. The study of flavonoids has attracted a lot of attention since it has been found that some of them are beneficial against pathogenic microbes [6].

A serious global health concern today, antibiotic resistance has grown significantly in the last several years. Antimicrobial resistance in pathogenic bacteria and fungi, particularly in hospital settings, has resulted in an inability to treat or manage infectious diseases in a timely manner. Even with alternatives to antibiotics derived from natural sources, it is still necessary to look for safe and affordable options [6]. The World Health Organization (WHO) suggests implementing cost-effective strategies and funding to address antibiotic resistance because these are essential for knowledge generation and application [7].

Plants naturally produce flavonoids, which are phenolic compounds, as secondary metabolites. Their molecular structure is based on a C15 skeleton, which consists of two benzene rings connected by a C6-C3-C6 three-carbon chain. They can exist in a free state or as glycosides. The ingredients "Yuen-Hua" in Chinese medicine include genkwanin and apigenin, which may have anthelmintic and diuretic properties [8]. Preliminary findings from our previous studies indicate that these compounds are active against a limited number of tested organisms [9]. It is likely difficult to extract a significant quantity of the active ingredient from the plants or cells, which accounts for this. This investigation seeks to validate the antimicrobial and therapeutic properties of these compounds. Because of their strong activity against a range of microorganisms, the study's underlying premise is that naturally occurring (bioactive) compounds or metabolites from plants or microbes can be employed as novel antimicrobial compounds. The need to find alternative antimicrobial substances arises from the growing threat of antibiotic resistance. The present investigation examines the antimicrobial and mycotoxin-producing potential of four flavonoid compounds.

Fisetin has been found to have an impact on *Staphylococcus, Bacillus, Pseudomonas aeruginosa*, and *Escherichia coli*. Fisetin is derived from a variety of fruits, including strawberries, apples, mangoes, and more. Fisetinidin, a compound derived from the heartwood of *Acacia mearnsii*, has been shown to affect *Staphylococcus aureus*. Gingabranin, a plant derivative of *Piscidia piscipula*, was found to have an effect on *Streptococcus mutans *[10]. Tropoflavin, flavonolignan, and quercetin were found to have an effect on *Staphylococcus aureus *[11]. It has been demonstrated that the apigenin found in celeriac and chamomile teas affects both periodontopathogenic and cariogenic bacteria. Research has demonstrated the effects of flavonolignan, a derivative of milk thistle, on *Staphylococcus aureus *and *Pseudomonas aeruginosa *[12]. The effects of luteolin, a compound derived from vegetables, on *Staphylococcus aureus *and *Listeria monocytogenes *have been found. Studies on *Escherichia* *coli, Klebsiella pneumoniae, Helicobacter pylori, Pseudomonas aeruginosa*, and *Staphylococcus aureus *have demonstrated the effects of fruit-based naringenin [11]. *Listeria monocytogenes *was found to be impacted by dihydromyricetin (DHM), which is extracted from vine tea leaves [13]. Research has shown that the human diet's source of isoflavone has an impact on the bacteria *Bacteroides fragilis, Bacteroides thetaiotaomicron, Slackia equolifaciens*, and *Lactobacillus delbrueckii *subsp. *bulgaricus*. Rumiponone, a methanol extract from *Paulownia tomentosa*, was found to have an effect on *Staphylococcus epidermidis *[14].

Fisetin has been observed to have an effect on *Escherichia coli, Bacillus *sp.*, Pseudomonas aeruginosa*, and *Staphylococcus *sp. Many fruits, such as mangoes, apples, strawberries, and more, are the sources of fisetin. Fisetinidin, which is isolated from the heartwood of *Acacia mearnsii*, has been found to have an impact on *Staphylococcus aureus*. It was found that the plant *Piscidia piscipula *contains glabranin, which has an effect on *Streptococcus mutans *[11].

Quercetin, flavonolignan, and tropoflavin were found to have an effect on *Staphylococcus aureus *[12]. It has been found that the extract known as apigenin, found in chamomile and celeriac tea, has an effect on bacteria that is both cariogenic and periodontopathogenic. *Pseudomonas aeruginosa *and *Staphylococcus aureus *have been found to be impacted by the flavonolignan found in milk thistle [13].

The effects of luteolin, a compound derived from vegetables, on *Staphylococcus aureus *and *Listeria monocytogenes *have been found. The effects of naringenin, a fruit-based food additive, on *Klebsiella pneumoniae, Escherichia coli, Helicobacter pylori​​​​​​​, Pseudomonas aeruginosa, *and *Staphylococcus aureus *have been found [12]. It has been found that *Listeria monocytogenes *is impacted by DHM, which is extracted from vine tea leaves [14]. It was found that the substance isoflavone, which is obtained from the human diet, had an effect on *Bacteroides fragilis, Bacteroides delbrueckii *subsp. *bulgaricus, Bacteroides thetaiotaomicron*, and *Slackia equolifaciens*. Imulone, a methanol extract derived from *Paulownia tomentosa*, was found to have an effect on *Staphylococcus epidermidis *[15].

It was found that wogonin and diplacone, which are extracted from the Pacific Island plant *Scutellaria strigillosa*, had an effect on *Staphylococcus epidermidis *[16]. It has been found that *Enterococcus faecalis *is impacted by kaempferol, which is obtained from strawberries, apples, broccoli, beans, and ea. Catechin was found to have an effect on *Escherichia coli *and *Salmonella sp*. [17]. Flavonolignan and epigallocatehin-3-gallate have an impact on *Pseudomonas aeruginosa *and *E. coli *[18, 19]. Fruits, vegetables, berries, and teas with myricetin-3-glycosides have been found to have an effect on *Bacillus cereus, Escherichia coli *​​​​​​*, *and *Staphylococcus aureus*. It has been found that *Klebsiella pneumoniae*, *Escherichia coli, Helicobacter pylori, Pseudomonas aeruginosa, *and *Staphylococcus aureus *are all impacted by naringenin, which is extracted from edible fruits [11, 19].

Positive results have been obtained from the flavonoid in vitro analyses. Against *Candida albicans *infections in mouse models, the subclasses chalcones and flavonols exhibit strong in vivoantifungal activity. In a mouse model of vulvovaginal candidiasis, quercetin in particular has been given via vaginal gavage, and in mice that have been infected with *Candida albicans*, licochalcone A, also known as chalcone, has been shown to be used topically orally [20].

The present investigation examined relevant literature and examined the potential inhibitory actions of fisetin, fisetinidin, 7,3`,4`-trihydroxyflavone (THF), and 7,3`,4`-trihydroxyflavonol (THF-) against particular pathogenic microorganisms.

## Materials and methods

Study design

The present study was an observational, laboratory-based, in vitro experimental investigation with a review of the current literature on the antimicrobial effects of flavonoids. The paper aimed to explore the inhibitory properties of four flavonoid compounds (fisetin, fisetinidin, THF, and THF-) against selected pathogenic bacteria and fungi. The assumption was that these flavonoid compounds would show antimicrobial activity against a variety of gram-positive, gram-negative, and fungal organisms. The independent variables were the four flavonoid compounds at differing concentrations (100-1000 μg/mL). The dependent variables were the growth inhibitions of the 19 microbial organisms tested.

Research plan

The Department of Microbiology and Clinical Parasitology, King Khalid University College of Medicine, conducted this study between 2021 and 2024. The King Khalid University College of Medicine's Ethics Committee granted ethical approval (REC # 2017-02-17). No personally identifiable information about the patients was provided by this study. Isolates of bacteria and yeast, including the quality control species, were taken from our laboratory collection.

Flavonoids as compounds

**Figure 1:**
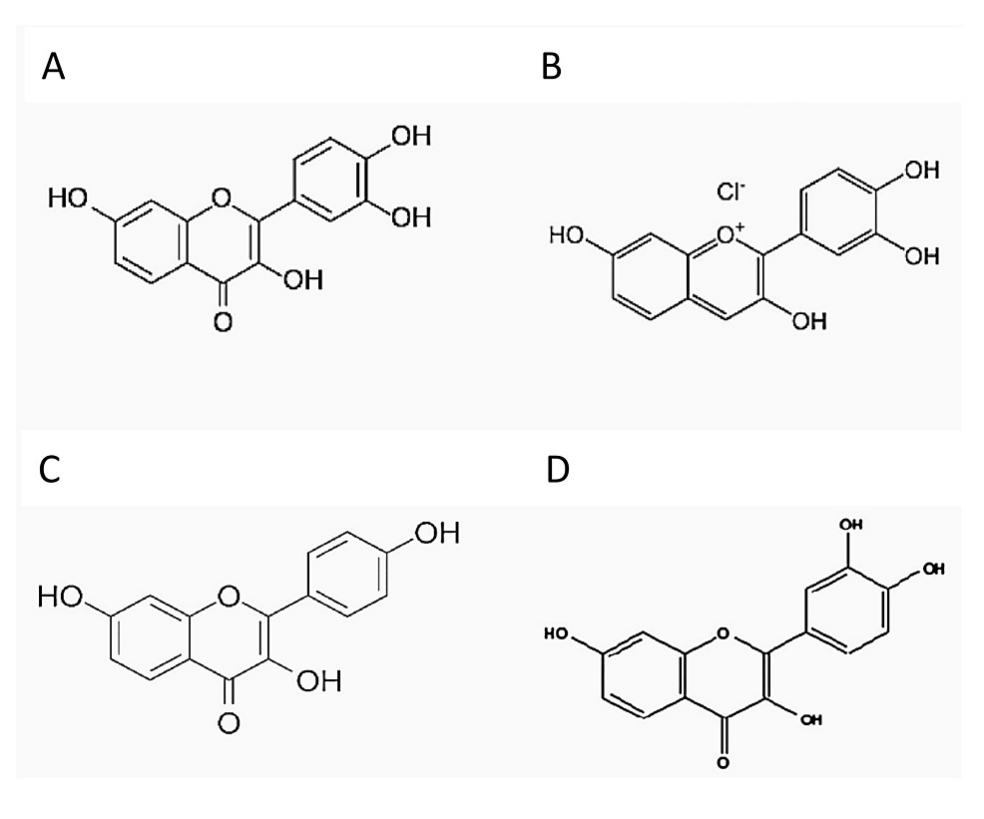
Structures of the flavonoid compounds used in this study A, fisetin; B, fisetinidin; C, 7,3`,4`-trihydroxyflavone (THF); and D, 7,3`,4`-trihydroxyflavonol (THF-) Figure created using PubChem (https://pubchem.ncbi.nlm.nih.gov/); PubChem National Center for Biotechnology Information, U.S. National Library of Medicine, Bethesda, MD.

BOC Sciences (BOC Sciences, Shirley, NY; www.bocsci.com) provided the compounds fisetin and fisetinidin. As previously mentioned [9], 7,3`,4`-trihydroxyflavonol was extracted from *Acacia nilotica *var. *adstringens*. Figure 1 displays a thorough explanation of the three compounds' structures. Before being needed, stock solutions (50,000 µg/mL) were made in water and refrigerated at -20 °C.

Organisms

Organisms representing major gram-positive, gram-negative, and yeast fungi (Table 1) were used to test the bioactivity of the three compounds. Organisms were subcultured to obtain fresh cell biomass. Subculturing was done using nutrient agar media (bacteria) and sabouraud dextrose agar (SDA) medium (yeast). Cultures were incubated at 37 °C for up to five days. Cultures are checked for purity before being processed further. *Staphylococcus aureus *(ATCC 25923) and *Pseudomonas aeruginosa *(ATCC 27853) were used as quality control strains.

**Table 1:** List of target microorganisms, diseases they cause, and resistance to antimicrobial agents* MRSA: methicillin-resistant *Staphylococcus aureus* [21,22]

Organism	Disease	Resistance to antimicrobials
Gram-positive bacteria		
Bacillus spp.	*Bacillus anthracis* is the causal agent of anthrax. Other *Bacillus spp.*, in particular Bacillus cereus and, to a lesser extent, *Bacillus subtilis* and *Bacillus licheniformis*, are occasionally linked with bacteremia/septicemia, endocarditis, meningitis, and infections of wounds, the ears, eyes, respiratory tract, urinary tract, and gastrointestinal tract.	*Bacillus spp.* were commonly resistant to β-lactam, glycopeptide, and sulfonamide antibiotics, including cefixime, penicillin G, cefuroxime, ceftriaxone, vancomycin, amoxicillin (62.3%), erythromycin, nitrofurantoin, and tetracycline.
Corynebacterium spp.	*Corynebacterium spp.* are normal microflora of the human body in addition to playing a role in the development of diseases in both immunocompromised and immunocompetent patients, mainly in the upper respiratory tract.	*Corynebacterium spp.* usually displays resistance to antimicrobial drugs with oral bioavailability and is related to the increased application of parenteral antimicrobial drugs.
Staphylococcus spp.	*Staphylococcus spp.* are the principal cause of skin and soft tissue infections, bloodstream infections, pneumonia, or bone and joint infections.	Almost all over the world, the community MRSA strains are dominating the routine isolate of *Staphylococci aureus*.
Streptococcus spp.	*Streptococcus spp.* are prominent causal agents of a variety of infections, notably sinusitis, otitis media, pneumonia, bacteremia, osteomyelitis, septic arthritis, and meningitis.	Worldwide, resistance acquisition in streptococci is slower compared to that in Enterobacteriaceae. This is possibly owing to the limited horizontal spread of resistance genes. Globally resistant streptococcal phenotypes have been described in streptococci isolated from animals.
Gram-negative bacteria		
Acinetobacter baumannii	*Acinetobacter baumannii* is a recognized agent of infections in the blood, urinary tract, and lungs (pneumonia), wounds, and other parts of the body.	Resistance to *Acinetobacter baumannii* causes a considerable threat to severely ill patients in intensive care units. Carbapenem-resistant *Acinetobacter baumannii* was ranked in 2018 by the WHO as the number one urgent need for antibiotic study and research.
Escherichia coli	*Escherichia coli* is a well-known agent of many enteric infections, including urinary tract infections, abdominal and pelvic infections, pneumonia, bacteremia, and meningitis.	*Escherichia coli *isolates could be 100% resistant to penicillin and erythromycin, then 98% to nalidixic acid, and less resistant to cephalexin, amoxicillin, ampicillin, ciprofloxacin, tetracycline, cefixime, and gentamicin.
Proteus vulgaris	Proteus species are commensals of the human gut that hold considerable enteric pathogenic potential. Proteus species is associated with Crohn's disease.	*Proteus vulgaris* is instinctively resistant to polymyxins (colistin), nitrofurans, tigecycline, and tetracycline.
Pseudomonas aeruginosa	*Pseudomonas aeruginosa* is a causal agent for a broad range of infections with variable severity. It is identified as causing nosocomial infections and ventilator-associated pneumonia, mainly affecting immunocompromised individuals, severe burn victims, and patients with underlying health conditions such as cystic fibrosis and chronic obstructive pulmonary disease (COPD).	*Pseudomonas aeruginosa* isolates produce extended-spectrum β-lactamases (ESBLs), which are responsible for a high degree of resistance to the mainstream of β-lactam antibiotics, comprising penicillins, cephalosporins, and aztreonam.
Yeasts		
Candida spp.	*Candida spp.* have the ability to cause disease since they possess specific virulence factors. Also, the immune status of the host and the local environment allow *Candida spp.* to develop many mechanisms to avoid the host's immune defenses and start infection. Skin and soft tissue infections, vaginal, oral, and bloodstream infections are among the major types of infections caused by *Candida spp..*	The primary treatment option is amphotericin B, a drug that can be toxic for patients who are already very sick. Also, treatment choices remain very narrow if patients infected with *Candida *infections are found to be resistant to both fluconazole and amphotericin B.
Cryptococcus spp.	*Cryptococcus spp.* cause cryptococcosis by invasion and are commonly associated with immunosuppressive individuals. *Cryptococcus neoformans* and *Cryptococcus gatti* are the most common and rarely produce infections in healthy people.	In spite of treatment, the mortality rates connected with cryptococcosis are eminent. Treatment choice is limited given the fact that both amphotericin B and flucytosine are harmful, need intravenous administration, and are mostly inaccessible in low-income countries as their cost is high.

In vitro assay

The in vitro activity of the flavonoid compounds against gram-positive, gram-negative, and yeasts was done using the agar-well diffusion method [23]. On nutrient agar plates, bacterial and yeast strains were grown, and they were then incubated for 24 hours at 37°C. Standardization was applied to all bacterial isolates using 0.5 McFarland (1.5x108 CFU/mL). Using a sterile cotton swab, each isolate was inoculated into Mueller Hilton agar (MHA) medium, and the incubation period was 10 minutes. With a 0.5-cm-diameter cork borer, holes were made in the MHA medium. For every concentration (1000, 10,000, 100,000, and 1,000,000 µg/mL), four holes were created. 

The flavonoid components were dissolved in distilled water to give the desired concentrations: 100, 300, and 1000 µg/mL. After that, 100 µL of the extract was added to each well, and the wells were incubated at 37 °C for 24 hours. The positive controls for gram-positive bacteria were vancomycin 30 µg (Oxoid, Thermo Fisher Scientific Inc., Waltham, MA), gram-negative bacteria were imipenem 10 µg (Mast Diagnostics. Group Ltd, Merseyside, UK), and yeasts were amphotericin B 100 µg (Bio-Rad Laboratories, Inc., Hercules, CA). The negative control was a disc impregnated with normal saline. After a 24-hour incubation period at 37 °C, growth inhibition was noted.

## Results

**Figure 2:**
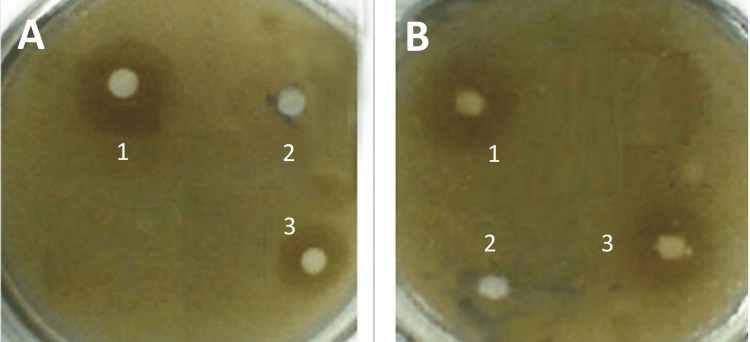
Inhibitory effect of 7,3`,4`-trihydroxyflavonol (THF-) on Bacillus cereus (A) and Staphylococcus aureus (B) disc 1: positive control (vancomycin, 30 µg); disc 2: THF- 300 µg/mL; and disc 3: THF- 1000 µg/mL) Image credit: ME Hamid

Table 2 displays the in vitro activity of the chosen flavonoid compounds against yeasts and gram-positive and gram-negative bacteria. The results showed the flavonoid compounds' inhibitory zones, indicating the presence of antibacterial activity against most of the tested organisms (Figure 2).

**Table 2:** In vitro antibacterial and antifungal effects of flavonoid compounds against gram-positive, gram-negative, and yeasts ATCC: American Type Culture Collection; T: type strain; *: clinical isolate; ND: not done; MRSA: methicillin-resistant *Staphylococcus aureus*

Organism	Compound (concentration)			
	Fisetin	Fisetinidin	THF (7,3, 4`-trihydroxyflavone)	THF- (7,3`,4`-trihydroxyflavonol)
Gram-positive bacteria				
Bacillus cereus*	ND	ND	Inhibited (300 µg)	Inhibited (1000 µg)
*Bacillus subtilis *(ATCC 6633)	Inhibited (100 µg)	Inhibited (100 µg)	ND	ND
*Corynebacterium diphtheriae *(ATCC 13812)	ND	ND	Inhibited (300 µg)	Inhibited (1000 µg)
*Enterococcus faecalis* (ATCC 19433^T)^	Inhibited (300 µg)	Inhibited (300 µg)	Inhibited (300 µg)	Inhibited (1000 µg)
*Staphylococcus aureus* (ATCC 25923)	Inhibited (300 µg)	Inhibited (300 µg)	Inhibited (300 µg)	Inhibited (1000 µg)
*Staphylococcus aureus* (MRSA)	Inhibited (100 µg)	Inhibited (100 µg)	ND	ND
Staphylococcus aureus*	Inhibited (300 µg)	Inhibited (300 µg)	Inhibited (300 µg)	Inhibited (1000 µg)
*Streptococcus agalactiae *(ATCC 13813^T^)	ND	ND	Inhibited (300 µg)	Inhibited (1000 µg)
Staphylococcus epidermidis	Inhibited (100 µg)	Inhibited (100 µg)	ND	ND
Gram-negative bacteria				
*Acinetobacter baumannii *(ATCC 19606^T^)	Inhibited (500 µg)	Inhibited (500 µg)	Inhibited (300 µg)	Inhibited (1000 µg)
*Acinetobacter *sp. *	Inhibited (500 µg)	Inhibited (500 µg)	Inhibited (300 µg)	Inhibited (1000 µg)
Escherichia coli *	Not Inhibited (500 µg)	Not Inhibited (500 µg)	ND	ND
*Escherichia coli* (Ec4662*)	Not Inhibited (500 µg)	Not Inhibited (500 µg)	Inhibited (300 ug)	Inhibited (1000 ug)
*Proteus vulgaris* *	Inhibited (500 µg)	Inhibited (500 µg)		
*Pseudomonas aeruginosa* *	Inhibited (500 µg)	Inhibited (500 µg)	ND	ND
*Pseudomonas aeruginosa* (ATCC 27853)	Inhibited (500 µg)	Inhibited (500 µg)	Inhibited (300 µg)	Inhibited (1000 µg)
Yeasts				
*Candida albicans *(ATCC 10231)	Inhibited (500 µg)	Inhibited (500 µg)	Inhibited (300 µg)	Inhibited (1000 µg)
*Candida albicans* (VY4661*)	Inhibited (300 µg)	Inhibited (300 µg)	ND	ND
*Cryptococcus neoforman*s (VY4662*)	Inhibited (300 µg)	ND	Inhibited (300 µg)	Inhibited (1000 µg)

*Bacillus cereus *was inhibited by both THF (300 µg) and THF- (1000 µg). On the other hand, fisetin (100 µg) and fisetinidin (100 µg) inhibited *Bacillus subtilis*. The inhibition zones are shown clearly in Figure 2 and Table 2.

*Corynebacterium diphtheriae *was inhibited by both THF (300 µg) and THF (-1000 µg). *Enterococcus faecalis *and *Staphylococcus aureus *were inhibited by fisetin (100 µg), fisetinidin (100 µg), THF (300 µg), and THF- (1000 µg). Fisetin (100 µg) and fisetinidin (100 µg) both inhibited *Staphylococcus epidermidis*.

Fisetin (500 µg), fisetin (500 ug), THF (300 µg), and THF- (1000 µg) all inhibited* Acinetobacter baumannii *and *Pseudomonas aeruginosa*. *E. coli *was resistant to fisetin (500 µg) and fisetinidin (100 µg), but not to THF (300 µg) or THF-1000 µg. Both 500 µg and 500 µg of fisetin inhibited *Proteus vulgaris*.

Fisetin (500 µg), fisetin (500 µg), THF (300 µg), and THF- (1000 µg) all inhibited *Candida albicans*. *Cryptococcus neoformans *was inhibited by 300 µg of fisetin but not by fisetinidin. Further suppression was demonstrated by 300 and 1000 µg of THF.

## Discussion

The present study has reviewed and compared the antibacterial properties of numerous flavonoids from different published sources with our current findings. Our results are supported by the fact that many of these substances have shown inhibitory effects on a variety of gram-positive and gram-negative bacteria, as well as yeast organisms [16].

Rich sources of flavonoids include wine, propolis, tea, honey, nuts, seeds, fruit, vegetables, stems, and flowers. They are also widely distributed within photosynthesizing cells. For centuries, the main physiologically active components of drugs used to treat illnesses in humans have been these compounds. An increasing amount of research is being done on this class of natural compounds in relation to anti-infectives. Antifungal, antiviral, and antibacterial flavonoids have been found, and their structures have been described by a number of groups [10]. Flavonoids are useful in medicine because conventional medicine can effectively use preparations that contain these physiologically active ingredients. For instance, traditional medicine in Argentina frequently employs *Tagetes minuta*, which contains quercetetagetin-7-arabinzylgalactoside, to treat a variety of infectious diseases [24]. Among other substances, ligoflavone C and derrone were found in *Retama raetam* (Forssk) flower extracts, and these compounds showed antibacterial activity against both gram-positive and gram-negative bacteria [25]. *Tripleurospermum disciforme*, popularly called "mayweed," is a plant that is rich in flavonoids, such as apigenin, kaempferol, luteolin, and quercetin, as well as the glycosides that go along with them. It is used as a disinfectant and to treat a variety of illnesses in traditional Iranian medicine [26].

The results of this study suggested that recently synthesized and little-studied compounds might find application in the treatment or prevention of infections through infection prevention. When compared to well-known antimicrobial drugs like metronidazole, the molecules THF, fisetin, and fisetinidin are effective antimicrobials. Our findings align with previous research findings. Our previous study [9] showed that these compounds are effective against the organisms under test. Fisetin was found to have an effect on *Escherichia coli, Bacillus, Pseudomonas*, and *Staphylococcus *species [27]. Similarly, fisetinidin from *Acacia mearnsii* heartwood was found to have an effect on *Staphylococcus aureus *[28]. A common metric used to describe antimicrobial flavonoids is their minimum inhibitory concentration (MIC), or the lowest concentration at which bacterial growth is noticeably inhibited. Evaluating novel antimicrobials typically starts with a MIC assessment. Assays involving the dilution of broth or agar are used to determine it. Promising compounds include purified substances with less than 10 µg/mL MIC and plant extracts with less than 100 µg/mL MIC [29].

According to the findings of the dental plaque microbial community, apigenin exhibited activities against *Staphylococcus aureus *and *Enterococcus faecalis*, while luteolin, morin, naringin, quercetin, and rutin inhibited *Actinomyces naeslundii, Actinomyces viscosus, Aggregatibacter actinomycecomitans, Enterococcus faecalis*, and *Escherichia*​​​​​​* coli*. The only bacteria that showed catechin's antibacterial properties were *Aggregatibacter actinomycetemcomitans* and *Staphylococcus aureus*. Moreover, luteolin, naringin, morin, quercetin, and rutin were shown to inhibit *Candida albicans *[4].

Pathogenic strains of various species, including *Staphylococcus aureus, Enterococcus faecalis, Actinomyces*, and *Escherichia* *coli*, are particularly dangerous to medical practice because they are resistant to drugs [30]. The creation of novel antibiotics has taken precedence over the treatment of these infections, as there are currently no antibiotics available. Scientists have been searching for edible and safe compounds that could prevent the growth of cariogenic biofilms in the past few years. Proanthocyanidins derived from cranberries have been shown to possess the ability to avert dental caries by hindering *Streptococcus mutans *and *Streptococcus sobrinus *from synthesizing organic acids [31].

Flavonoids exhibit a wide range of modes of action, as evidenced by their ability to disrupt membranes, influence the formation and growth of bacterial biofilms and the corresponding bacterial adhesion, inhibit the synthesis of cell envelopes, nucleic acids, electron transport chains, and adenosine triphosphate (ATP), and inhibit bacterial toxins. Many flavonoids have so far been identified due to their efficacious antibacterial properties in the fight against plant infections. These characteristics can be successfully expanded to fight infections that affect humans. Furthermore, several plant-based flavonoids have antibacterial properties that differ from those of traditional medications; as a result, they may be useful in enhancing antibacterial treatment [32].

Strengths of the study

This study offers an important new insight into the antimicrobial properties of flavonoids and their effectiveness against popular bacterial and fungal pathogens. The comprehensive literature review also offers a valuable synthesis of the up-to-date state of research in this field. This study's inference that flavonoids, which are simply reachable food elements, are still an effective treatment for infectious illnesses is one of its main strengths. As stated by the research, several flavonoids, such as fisetin, fisetinidin, THF, and THF- might be helpful in stopping the growth of some infections, including staphylococci. Furthermore, improvements in the pharmacokinetics and diffusion of flavonoids may facilitate their adoption as a therapeutic adjunct to traditional medications.

Study limitations

A potential constraint of the research is the qualitative measurement of the inhibitory activities of the four compounds, which may not be sufficient. To facilitate statistical analysis, no assay runs in duplicate or triplicate were carried out. One of the study's shortcomings is the small sample size of the species being studied, which makes it difficult to get more conclusive results. Furthermore, the MIC of the four medications has not been determined using the microdilution method.

Future recommendation

Future research should apply quantitative techniques to more accurately ascertain the minimal inhibitory doses of the flavonoid compounds against a larger panel of microbiological species that are clinically relevant in order to build on these findings. Potential therapeutic uses for these flavonoids may be revealed by assessing how well they combine with traditional antibiotics.

## Conclusions

To sum up, the findings of this study demonstrate that flavonoids that are found naturally have antibacterial properties. To determine the role of flavonoids in disease control, more research must be done. It may be useful in treating a number of infections, particularly skin and upper respiratory infections if used in appropriate pharmaceutical formulations. Any improvements in its pharmacokinetics and diffusion would favor its introduction as an adjuvant treatment in addition to conventional agents. These compounds could be made into drugs to fight dangerous bacteria and fungi that are resistant to antibiotics.
